# Environmental Monitoring and Analysis of Persistent Organic Pollutants

**DOI:** 10.3390/toxics11060535

**Published:** 2023-06-15

**Authors:** Vlasta Drevenkar, Gordana Mendaš

**Affiliations:** Institute for Medical Research and Occupational Health, HR-10000 Zagreb, Croatia; vdreven@imi.hr

Persistent organic pollutants (POPs) are a group of 28 toxic compounds of different chemical classes listed in the Stockholm Convention on POPs, which aims to protect the environment and human health. This group includes chemicals used in agriculture (pesticides), industry (e.g., polychlorinated biphenyls, polybrominated diphenyl ethers, perfluorinated compounds), as well as unintentional industrial or combustion by-products, such as polychlorinated dibenzo-*p*-dioxins and dibenzofurans. POPs are highly persistent, bioaccumulative and biomagnifying, since they resist chemical, biological, and photolytic degradation. They have been identified in air, water, soil/sediment, and biota all over the world, even in remote regions where they have never been used, not only as a consequence of local contamination sources, but also as a result of efficient long-distance transport by circulation of air masses and waters. POPs enter the aquatic environment through dry and wet atmospheric deposition and attach to soil particles in water runoffs or waste waters. Due to their tendency to sorb to suspended particulate matter, POPs accumulate in aquatic (marine, river, lake) sediments as one of their main sinks.

For the trace determination of POPs in complex environmental and biological matrices, highly selective and sensitive analytical methods are fundamental. Most of these methods are based on selective extraction of target compounds and analysis of purified extracts with high-resolution instrumental techniques, such as gas or liquid chromatography coupled to mass spectrometry.

This Special Issue includes six original research articles focused on (a) studies of origin, temporal and spatial distribution, and long-term trends of selected organic pollutants in different environmental compartments; (b) recent advances in analytical methods developed for their identification and quantification in environmental and biological matrices; and (c) prediction of pollutant levels and fate in the environment based on model simulation.

In their paper entitled “Distribution and Relationships of Polycyclic Aromatic Hydrocarbons (PAHs) in Soils and Plants near Major Lakes in Eastern China”, a team of Chinese researchers compare the composition and mass fractions of 16 PAHs in soil and plant samples collected close to the shore of nine lakes [1]. Based on diagnostic ratios of PAH mass fractions and principal component analysis, the authors identify combustion processes as the main source of PAHs both in soils and plants, discuss the relationships between individual PAHs in soil-plant systems, and compare the potentials of different plant species for phytoremediation of PAH-contaminated soil.

A team of Croatian researchers address PAH-related human health and ecological risks in the paper “Carcinogenic Activity and Risk Assessment of PAHs in Ambient Air: PM_10_ Particle Fraction and Bulk Deposition” based on toxic equivalence factors and equivalent benzo(a)pyrene (BaP) concentrations for PM_10_ particle and bulk deposition samples collected in a residential part of the Croatia’s capital Zagreb [2]. The authors assess potential cancer risk via ingestion, dermal contact, and inhalation for two different groups of Zagreb residents and rely on the risk quotient approach to assess possible ecological risk from PAHs in bulk deposition.

The article “Potential of Coupling Metaheuristics-Optimized-XGBoost and SHAP in Revealing PAHs Environmental Fate” by a team of Serbian researchers [3] describes the use of an optimized XGBoost model and the Shapley Additive exPlanations (artificial intelligence) method to analyse a set of two years’ worth of data on air pollutant measurements (PM_10_ atmospheric particles, particle-bound benzo(a)pyrene, Pb, As, Cd, Ni, gaseous pollutants NO, NO_2_, NOx, and SO_2_) and meteorological parameters. The authors distinguish types of environments characterized by specific interactions between benzo(a)pyrene, other polluting species, and meteorological conditions and explain how advanced artificial intelligence-based modelling can contribute to a better understanding of complex factors that determine the fate of air polluting species in the environment.

The fourth article presented in this issue describes in detail a new method for determination of flame retardants polybrominated diphenyl ethers (PBDEs) and hexabromocyclododecane (HBCDD) in different types of food and feed, developed by a group of Slovenian researchers (“Simultaneous Method for Selected PBDEs and HBCDDs in Foodstuffs Using Gas Chromatography—Tandem Mass Spectrometry and Liquid Chromatography—Tandem Mass Spectrometry”) [4]. The new method enables simultaneous determination of all nine relevant PBDE congeners (BDE-28, 47, 49, 99, 100, 153, 154, 183, and 209) and HBCDD isomers (alpha, beta, and gamma) in the same (food or feed) sample extract, which is first analysed with GC-MS/MS(EI) for PBDEs and then with LC-MS/MS(ESI) for HBCDDs. The limits of quantification for all PBDEs and HBCDDs are in line with the recommendations of the European Commission.

The fifth article, “Emerging Contaminant Imidacloprid in Mediterranean Soils: The Risk of Accumulation Is Greater than the Risk of Leaching”, brings the results of a Croatian-Polish collaboration in researching the behaviour of the neonicotinoid insecticide imidacloprid in Mediterranean soils [5]. Although this extensively used insecticide is not considered to be persistent, its frequent occurrence and retention in the environment is of concern worldwide. Its sorption/transport was investigated in five Croatian Mediterranean soils using the column experiments. The results indicate a higher affinity of imidacloprid to soil organic matter than to any other soil constituent. Additionally, they suggest an important contributing role of hematite, one of the main mineral constituents of Mediterranean terra rossa soils, to imidacloprid sorption. Consequently, the risk of imidacloprid accumulation in the soil is higher than the risk of contamination by short-term leaching. The authors conclude that future soil health protection programmes should include continuous monitoring of imidacloprid in soils.

The last article “Prediction of the Impact of Land Use and Soil Type on Concentrations of Heavy Metals and Phthalates in Soil Based on Model Simulation” by a team of Serbian researchers [6] looks into the possibilities of predicting soil pollution with an artificial neural network (ANN) model, more specifically a multi-layer perceptron (MLP) model of three layers (input, hidden, and output) based on the Broyden–Fletcher–Goldfarb–Shanno (BFGS) iterative algorithm. The results confirm good method predictivity and promising application in future studies of the relationship between soil properties and pollutant mass fractions.

We would like to thank all the authors for their valuable original scientific contributions to this Special Issue and the reviewers for their time and expertise. Our special thanks go to Ms Jessie Zang, the section managing editor, for her valuable suggestions and continuous support and help in promoting this Special Issue.
